# Prompt gamma imaging vs. range probing: Experimental comparison of two range verification approaches for proton therapy

**DOI:** 10.1002/mp.70142

**Published:** 2025-11-19

**Authors:** Stefanie Bertschi, Giuliano Perotti Bernardini, Jonathan Berthold, Jeffrey Free, Elisabeth Bodenstein, Gabriel Guterres Marmitt, Guillaume Janssens, Kristin Stützer, Stefan Both, Christian Richter

**Affiliations:** ^1^ OncoRay – National Center for Radiation Research in Oncology Faculty of Medicine Dresden Germany; ^2^ Department of Radiation Oncology University Medical Center Groningen University of Groningen Groningen The Netherlands; ^3^ Center for Advanced Systems Understanding, CASUS Görlitz Germany; ^4^ Helmholtz‐Zentrum Dresden‐Rossendorf CASUS ‐ Center for Advanced Systems Understanding Dresden Germany; ^5^ Helmholtz‐Zentrum Dresden ‐ Rossendorf Institute of Radiooncology ‐ OncoRay Dresden Germany; ^6^ Ion Beam Applications SA Louvain‐la‐Neuve Belgium; ^7^ Department of Radiotherapy and Radiation Oncology Faculty of Medicine and University Hospital Carl Gustav Carus TUD Dresden University of Technology Dresden Germany

**Keywords:** Cancer, experimental comparison, prompt gamma imaging, proton therapy, range probing, treatment verification

## Abstract

**Background:**

In online adaptive proton therapy (OAPT), additional treatment verification techniques are crucial for detecting treatment deviations and acting as a safety net, as phantom‐based patient‐specific quality assurance methods are not applicable. Prompt gamma imaging (PGI) has the potential for online treatment verification without adding dose nor prolonging treatment. PGI has proven to detect relevant anatomical changes under real‐world clinical conditions. Range probing (RP) has shown its clinical applicability for in‐vivo proton range assessment before treatment, thereby helping to address uncertainties. It has been proven useful for quality control of cone‐beam CT‐based synthetic CTs, suggesting its adoption into OAPT.

**Purpose:**

The performance of PGI and RP, two different yet complementary treatment verification methods, was compared by consecutive measurements within one experimental setup. Anatomical changes (AC) and setup errors (SE) were mimicked in an anthropomorphic head phantom.

**Methods:**

The PGI‐system was positioned beneath the phantom while the RP‐system was positioned distally, allowing a simultaneous setup of both systems at a horizontal gantry angle. A brain target was irradiated with a 1‐field pencil‐beam scanning treatment plan and a low‐dose RP‐plan with high‐energy protons that passed through the phantom. Upstream and downstream positioned water‐equivalent material slabs of 2/3/5 mm or 5 mm thicknesses mimicked AC within the beam path or beyond the target, respectively. Additionally, the couch was shifted 2 and 3 mm in left, down and upstream direction in beam's eye view (BEV), mimicking SE. Both plans were delivered and monitored 10 times for each AC and SE configuration. Geometrical range shifts measured with PGI were converted to water‐equivalent thickness range shifts for direct comparison between PGI and RP results.

**Results:**

Both systems detected range deviations relevant for the treatment field, caused by AC within the clinical beam path. RP measurements were more precise for all scenarios (RP: 1σ ≤ 0.3 mm, PGI: 1σ ≤ 0.8 mm). PGI was similarly accurate for the 2 mm slab while slightly underestimating 3 and 5 mm slabs (≤ 0.7 mm). Only RP detected AC beyond the target, which are irrelevant for the monitored treatment field.

Both systems detected expected range shifts of all SEs. RP was more accurate than PGI (RP: within 0.3 mm, PGI: within 0.8 mm). The couch movement left in BEV was detected with slightly higher precision using PGI (RP: 1σ ≤ 1.0 mm, PGI: 1σ ≤ 0.9 mm), while the precision of detecting the couch movement down in BEV was the same for both systems (1σ ≤ 0.7 mm). Only PGI recognized couch movements upstream (accuracy within 0.4 mm).

**Conclusion:**

Both PGI and RP precisely detected introduced AC relevant for the monitored treatment field. SE were detected, but with greater uncertainty. While the current implementation of RP enables pretreatment range verification after setup imaging with a low‐dose RP‐field, PGI enables treatment verification during field delivery, detecting range deviations relevant for the treatment field. The simultaneous setup of PGI and RP clearly demonstrated their compatibility in a clinical setting, the unique advantages of each system and their crucial role as safety nets in OAPT.

## 1 INTRODUCTION

Online adaptive proton therapy (OAPT) is a widely researched approach to overcome the limitations of conventional proton therapy, such as large uncertainty margins and restrictions in patient selection.^1^, ^2^, ^3^, ^4^ In OAPT, a treatment plan is adjusted to the patient's position and anatomy of the day, therefore improving the quality of a treatment plan by enabling steeper dose gradients and tighter margins. Because of time limitations with the patient at isocenter position, OAPT workflows can no longer accommodate established patient‐specific quality assurance (QA) measures.^1^ Consequently, there is an extra need for treatment verification techniques to detect unexpected treatment deviations and to offer an independent safety net. Prompt gamma imaging (PGI) and range probing (RP) — two different yet complementary treatment verification methods — are two of the most advanced techniques regarding their current clinical application in feasibility studies and both are highly promising tools for OAPT.

Prompt gamma treatment verification (PGTV) is based on prompt gamma (PG) radiation emitted during nuclear interactions of the proton beam within the patient. Hence, PGTV offers the potential for online in vivo treatment verification without adding dose to the patient or extending the treatment duration.^5^, ^6^, ^7^ Prompt gamma imaging (PGI), which leverages spatial information, has been under investigation at OncoRay since 2014, with its first clinical application implemented in 2015.^8^ A second‐generation, trolley‐mounted PGI system with improved positioning accuracy is currently used in an ongoing observational clinical study with more than 470 treatment field deliveries monitored so far. With the generated real‐world data, the ability of PGI to reliably detect clinically relevant anatomical changes has been proven.^9^ Currently, a clinical interventional study is in preparation, where PGI is applied prospectively to directly trigger a treatment intervention, namely a prompt acquisition of a control CT and subsequent treatment adaptation. Recent advancements in the PGI workflow have reduced the evaluation time for a treatment field to approximately 5 min, thereby enabling online evaluation and providing the basis for this interventional study.^10^ This approach will enable tighter safety margins.^11^ Furthermore, a recent study has shown that PGI is also a promising treatment verification tool for cone‐beam CT (CBCT)‐based OAPT.^12^

Range probing (RP), a specific form of proton radiography, is another method for verifying the range of protons^13^, ^14^, ^15^, ^16^ which has been applied at the University Medical Center Groningen (UMCG).^17^, ^18^ RP has been proposed as a quality control (QC) tool to perform in vivo proton range verification. Farace et al. introduced a method for detecting setup errors in patients, where residual ranges of single proton spots within an RP‐field are measured using a multi‐layer ionization chamber (MLIC) positioned at the beam exit.^14^ The first clinical implementation of RP was done for head‐and‐neck cancer patients at UMCG in 2020^19^ and is now accessible for conducting patient‐specific range accuracy checks as part of an in vivo RP‐QC procedure. It has been shown to provide added value by assessing the CT number accuracy in CBCT‐based synthetic CT (sCT) images, which could be beneficially introduced into OAPT workflows.^20^, ^21^, ^22^

Since the performance of treatment verification methods can depend on specific experimental settings and beam characteristics, the aim of our study was to analyze and compare range probing and prompt gamma imaging in a joint experiment conducted at OncoRay under equal conditions.

## 2 METHODS AND MATERIALS

PGI and RP were analyzed and compared for their ability to detect range deviations across 10 different simplified scenarios that could potentially occur during proton therapy treatments. The experiment was performed with an anthropomorphic head phantom (Proton Therapy Dosimetry Head, model 731‐HN, CIRS, Norfolk, Virginia, U.S.) at OncoRay.

### 2.1 Prompt gamma imaging system

The PGI system at OncoRay is mounted on a trolley, which is positioned underneath the patient table and fixed by a docking station, ensuring a highly reproducible position relative to the isocenter (1σ = 0.5 mm).^5^, ^8^, ^23^ The PGI camera consists of a tungsten knife‐edge slit collimator which projects the emitted PG radiation along the beam axis onto its spatially resolved detector.^5^ The detector comprises two rows of 20 lutetium–yttrium oxy‐orthosilicate scintillation crystals, each slab having dimensions of 4 × 31.5 × 100 mm^3^. These crystals are linked in a 1:1 configuration to silicon photomultipliers and the corresponding custom readout electronics.^24^ The recorded one‐dimensional PGI signal is compared to a reference signal to determine the proton range shifts inside the patient. These range shifts are measured in millimeters and represent a geometrical shift within the patient, therefore showing the direct impact of the anatomical change (AC) or setup error (SE) on the proton range.

### 2.2 Range probing system

The RP system used in this study consisted of a Giraffe MLIC detector (IBA Dosimetry GmbH, Schwarzenbruck, Germany) mounted on a trolley next to the patient table. The detector comprises 180 air‐vented plane‐parallel ionization chambers with a 2 mm spacing behind an entrance window with a 120 mm diameter. The nominal range accuracy is ± 0.5 mm.^13^, ^16^

The concept uses single proton pencil beams with sufficient energy to completely penetrate the anthropomorphic head phantom and be detected on the other side. The MLIC detector captures the proton pencil beams, specifically recording the integral depth dose curves (IDD) of the resulting Bragg peaks. By knowing the initial energy and depth of the pencil beam in water, the residual range of the protons is calculated, enabling the determination of the water‐equivalent thickness (WET) of the material traversed by the protons.^25^

### 2.3 Phantom data and treatment fields

The anthropomorphic head phantom was positioned upright on the patient's table to enable the delivery of the required gantry angles using a horizontal beam. A sample target (29.2 cm^3^), based on a clinical case, was delineated in the dorsolateral right brain region on a dual‐energy CT scan of the anthropomorphic head phantom.^26^, ^27^ The dual‐energy CT scan with 80 kVp and 140 kVp had a voxel size of 0.75 mm x 0.75 mm x 0.5 mm and the DirectSPR approach was used to derive the voxel‐wise stopping power information.^28^

#### 2.3.1 Treatment field

With PGI, range deviations were measured during the delivery of clinical treatment plans. The same pencil‐beam scanning (PBS) treatment plan with one horizontal field (270°) at 0° couch angle delivering $D_{\mathrm{RBE}}=$ 1 Gy, as employed by Hueso‐González et al.^27^ was used. A constant RBE of 1.1 was applied and a range shifter of 73.8 mm water‐equivalent thickness was used. The plan consisted of 779 spots distributed over 15 energy layers of 115–160 MeV.

#### 2.3.2 Range probing field

**FIGURE 1:**
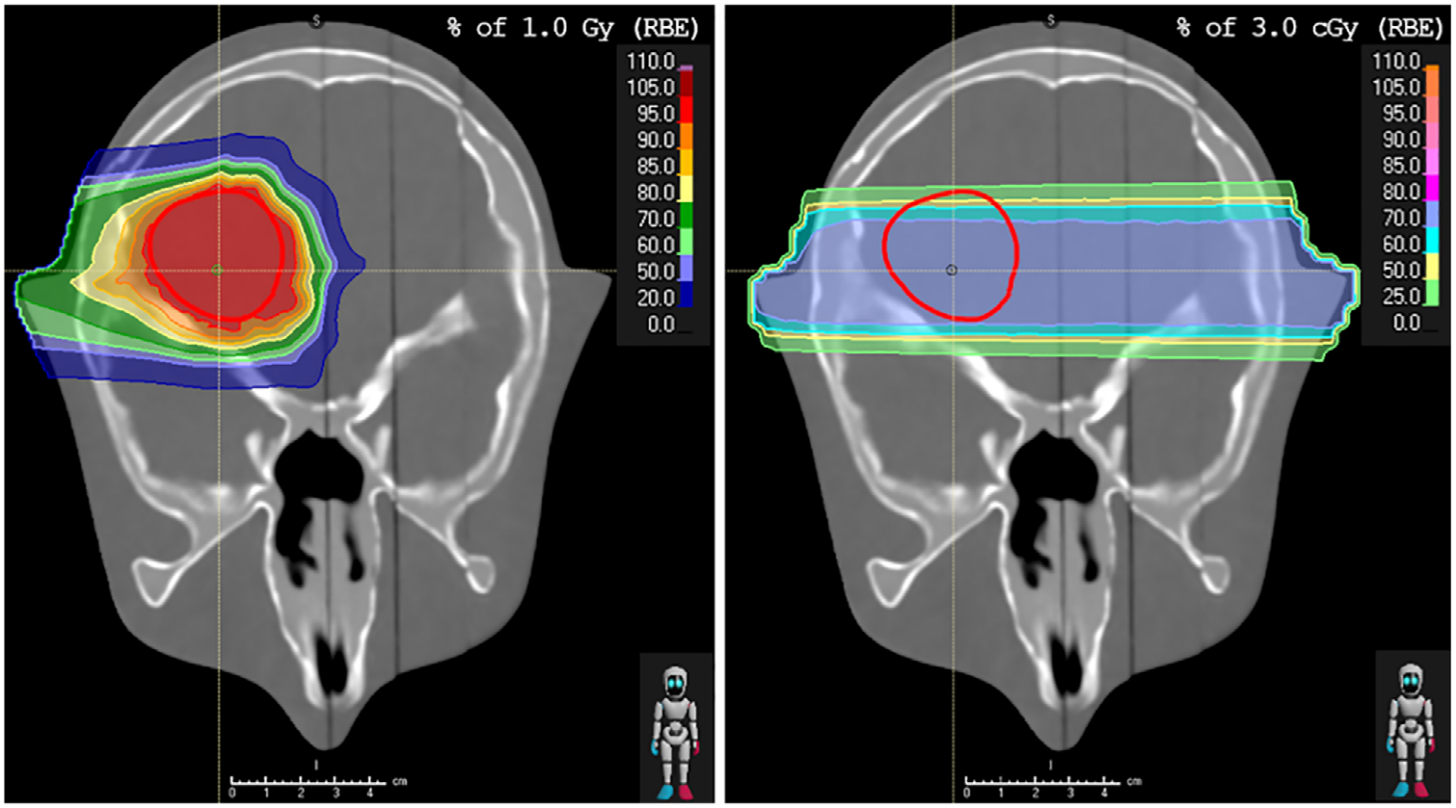
Treatment plan (left), used for PGI measurements, and RP‐plan (right) for the anthropomorphic head phantom with the target delineated in the dorsolateral right brain region (red).

RP measurements were performed using a dedicated horizontal (270°) RP‐field at 0° couch angle before the delivery of clinical treatment plans. The RP‐field covered a 4 x 4 cm^2^ square area around the isocenter of the treatment plan and consisted of 81 (9 x 9) spots (5 mm spot spacing) with 210 MeV energy. The RP‐field fully covered the target. The lowest monitor units (MU) accepted by the machine (0.04 MU, 6.2*10^6^ protons) were assigned to each spot, resulting in approximately 3 cGy of RBE‐weighted dose per RP‐plan. Both plans are shown in Figure 1.

### 2.4 Experimental setup

**FIGURE 2:**
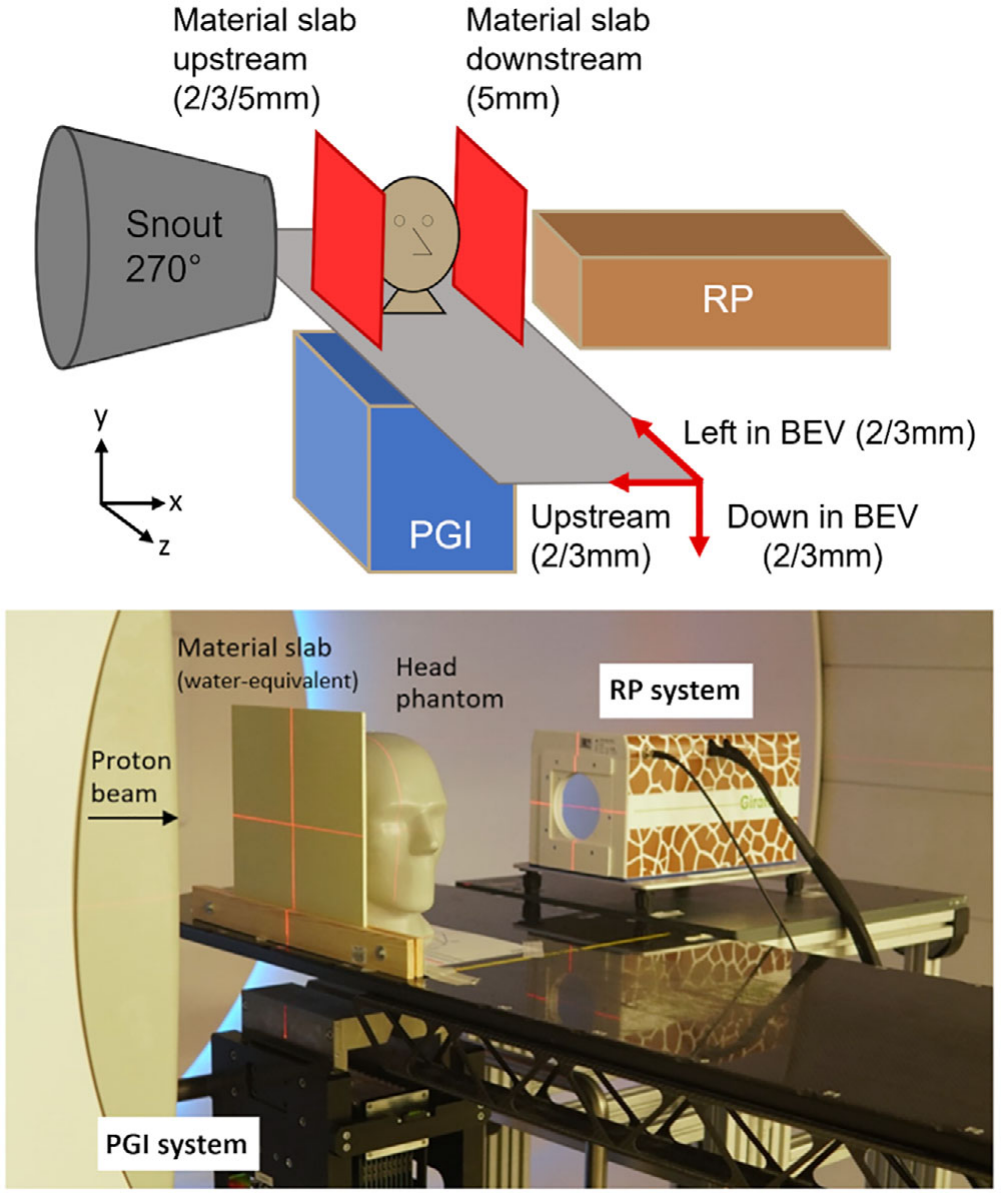
Schematic overview (top) of 10 investigated scenarios (red): Four scenarios simulated anatomical changes by introducing material slabs upstream or downstream of 2, 3, and 5 mm WET or 5 mm WET. Six scenarios simulated setup errors by introducing 2 or 3 mm couch movements either left in BEV, down in BEV or upstream. Experimental setup of both verification systems (bottom). BEV = beam's eye view, PGI = prompt‐gamma imaging, RP = range probing, WET = water‐equivalent thickness.

The PGI system was positioned underneath the patient table, while the RP system was positioned next to it, allowing a simultaneous setup of both systems (Figure 2). A total of 10 scenarios leading to different range deviations of the planned dose distribution were investigated (Table 1). Different water‐equivalent material slabs (Stopping power ratio (SPR) = 1) from CIRS (Sun Nuclear, Norfolk, Virginia, U.S.) of 2, 3, and 5 mm thickness were positioned upstream to the phantom to mimic AC within the beam path, therefore affecting the range within the target for those scenarios. For one scenario, a water‐equivalent material slab of 5 mm was positioned downstream to the phantom to mimic AC beyond the target, not affecting the range within the target for the considered treatment field. Additional scenarios simulated SE by moving the couch 2 or 3 mm in one of three axial directions (down in BEV, left in BEV, and upstream). For the SE scenarios, there were no material slabs added. The clinically realistic treatment plan and the RP‐plan were delivered and monitored 10 times in the undisturbed reference scenario and 10 times for each of the 10 scenarios with introduced AC or SE. All scenarios with introduced AC / SE errors were compared to the measurement of the reference scenario (i.e., no material slabs added and the couch in its initial position).

**TABLE 1:** Overview of the ten investigated treatment deviation scenarios and the expected range shifts in the target.

Scenario	Expected range shift in target^¹^
(1) Slab upstream (2 mm)	Slab thickness: 2 mm
(2) Slab upstream (3 mm)	Slab thickness: 3 mm
(3) Slab upstream (5 mm)	Slab thickness: 5 mm
(4) Slab downstream (5 mm)	0
(5) Couch movement left in BEV (2 mm)	Complex change requires independent dose calculation for shifted phantom position
(6) Couch movement left in BEV (3 mm)	
(7) Couch movement down in BEV (2 mm)	
(8) Couch movement down in BEV (3 mm)	
(9) Couch movement upstream (2 mm)	Close to 0
(10) Couch movement upstream (3 mm)	

^1^ Numbers are stated in water equivalent thickness.

### 2.5 Evaluation

For both treatment verification systems, measurements from the AC or SE scenarios were compared to those of the undisturbed reference scenario in the initial position without material slabs. Range shifts were extracted for each spot of the PBS‐treatment plan with PGI, called PGI range shifts, and for each spot of the RP‐plan, called RP range shifts. Measured range shifts for both systems were compared to the ground truth expectation. In case of AC, this was the thickness of the introduced slabs, and in case of the SE, the ground truth was derived from a dose recalculation for the shifted phantom position and extraction of the range deviation relative to the reference scenario (Table 1).

#### 2.5.1 Prompt gamma imaging

##### Data processing

For the PGI measurement of the reference scenario (no introduced change), data of ten 1 Gy measurements were accumulated, providing smooth profiles with high statistics. This resulted in one reference PGI profile for each spot.

For each range deviation scenario, 10 measurements were performed and separately compared to the reference scenario yielding 10 PGI range shifts for each spot. Following the procedure for PGI data acquired in clinical treatments,^9^ PGI range shifts were determined with a least‐square matching algorithm after aggregation of the spot‐wise PGI profiles to minimize statistical uncertainties by applying a two‐dimensional Gaussian smoothing (*σ* = 7.8 mm) for each layer. Specific spot filters were applied to include only spots within the field‐of‐view (FOV) of the camera and a minimal number of delivered protons of 0.5 x 10^8^ to ensure statistical reliability. Hence, 596 of the 779 spots were used for evaluation.

##### Independent dose calculation

Independent dose calculations (Monte Carlo dose engine v4.4) were performed at OncoRay in RayStation 9A (RaySearch Laboratories, Stockholm, Sweden) for all scenarios.^9^ Integrated depth‐dose (IDD) profiles were calculated for each pencil beam spot of each scenario. Spot‐wise IDD‐based range shifts were extracted by comparing the scenarios with introduced AC or SE to the reference scenario by also applying a least‐square matching algorithm (fall‐off match) after the same in‐layer 2D‐Gaussian (*σ* = 7.8 mm) aggregation. This provides a spot‐wise expectation of the range deviation for introduced AC and SE.^9^ This simulated expectation was used two‐fold. For the introduced SE, it served as ground truth range shifts for comparison with the PGI measurements. For the introduced AC, it was used to transform geometrical shifts measured by PGI into WET shifts measured with RP, as described in the next paragraph.

##### Transformation of geometrical range shifts into WET range shifts

PGI range shifts represent geometrical shifts in millimeters within the patient, while RP range shifts represent WET range shifts in millimeters.^14^, ^27^ Those measures are only equivalent if treatment spots are set in water, otherwise, the geometrical shift measured with PGI depends on the SPR value of the tissue the beam stops in. To enable a direct comparison of PGI and RP, measured PGI range deviations were transformed to WET range deviations as measured by RP. Multiplying the retrieved geometrical range shift ($S_{\mathrm{geom}})$ with the mean SPR value of tissue affected by the shift of a single Bragg peak ($\mathrm{SP}R_{\Delta R}$) leads to the retrieved WET shift ($S_{\mathrm{WET}}$).

(1) $$S_{\mathrm{WET}}=S_{\mathrm{geom}}\cdot \mathrm{SP}R_{\Delta R}$$

For the AC scenarios (introduced slabs), the WET shift of introduced water‐equivalent material slabs ($S_{\mathrm{WET},\mathrm{slab}}$) as well as the corresponding geometrical range shift of the independent dose calculation ($S_{\mathrm{geom},\mathrm{calc}}$) is known. Therefore, the mean SPR value of the affected tissue was calculated as follows:

(2) $$SPR_{\Delta R}=S_{\mathrm{WET},\mathrm{slab}}/S_{\mathrm{geom},\mathrm{calc}}$$

For those scenarios, the measured geometrical range shift of each spot ($S_{\mathrm{geom},\mathrm{meas}}$) was transformed directly into the measured WET range shift ($S_{\mathrm{WET},\mathrm{meas}}$) by multiplying it with the calculated $\mathrm{SP}R_{\Delta R}$.

(3) $$S_{\mathrm{WET},\mathrm{meas}}=S_{\mathrm{geom},\mathrm{meas}}\cdot \mathrm{SP}R_{\Delta R}$$

In case of the setup scenarios (couch movements), only the geometrical range shift is known from the dose calculation, but the shift in WET is theoretically unknown. However, since the target is located in homogeneous brain tissue, small couch movements affected the mean SPR value in the region of the Bragg peak shift only marginally (< 0.05%). All measured results obtained as geometrical range shift ($S_{\mathrm{geom},\mathrm{meas}})$ were transformed to a WET range shift ($S_{\mathrm{WET},\mathrm{meas}}$) by multiplication with the average SPR value of the brain region ($\mathrm{SP}R_{\mathrm{brain}}=1.04$).

(4) $$S_{\mathrm{WET},\mathrm{meas}}=S_{\mathrm{geom},\mathrm{meas}}\cdot \mathrm{SP}R_{\mathrm{brain}}$$

#### 2.5.2 Range probing

##### Data processing

For the evaluation, all 81 spots of the RP‐field were used. Each spot was analyzed independently, resulting in 81 measured IDD curves per RP‐plan delivered. For each scenario (reference scenario and 10 range deviation scenarios), the RP‐plan was delivered and monitored 10 times. To calculate the spot‐wise RP‐range shifts across the 10 scenarios with introduced AC or SE, we compared each measurement in the modified scenario with the corresponding measurement in the undisturbed reference scenario, that is, the first measurement of the modified with the first measurement of the reference scenario, and so on up to the 10^th^ measurement. This approach allowed us to generate ten 2D range shift maps (RSM) that captured the RP range shifts between the reference and modified curves for each evaluated scenario (similar to the PGI evaluation).

The 10 measurements in the reference scenario were performed to match the conditions used for PGI and to minimize beam delivery uncertainties. While PGI benefits from higher statistics by summing profiles for accurate reference measurements, a single RP measurement provides sufficient statistical accuracy by directly monitoring individual pencil beams.

RP shifts were determined by aligning the IDD curve of the reference scenario with that of the modified scenarios using the least squares method^14^ through the Open‐REGGUI toolbox, a MATLAB‐based platform for adaptive proton therapy.^29^

##### Independent dose calculation

Independent dose calculations (Monte Carlo dose engine V5.3) were performed at UMCG in RayStation 11B (RaySearch Laboratories, Stockholm, Sweden) for the reference and all modified scenarios. IDD curves were calculated for each pencil beam spot of the RP‐field. Spot‐wise 2D RP range shift maps were computed by comparing the scenarios with introduced AC or SE to the reference scenario, applying the same least‐square method^14^ in the Open‐REGGUI toolbox as for the measurements.^29^ This independent dose calculation served as the ground truth for expected RP range shifts when introducing SE.

#### 2.5.3 Direct comparison and ground truth definition

For both systems, the median range shift was computed for each spot of the respective plan (596 spots for PGI, 81 spots for RP) by comparing the 10 measurements for each AC and SE configuration with the undisturbed reference scenario. For all 10 investigated scenarios with introduced AC or SE, PGI and RP measurements were compared directly on the level of WET range shifts.

For introduced material slabs mimicking AC upstream to the phantom, we expected to measure the introduced WET shift of the material slabs with both the PGI and the RP systems. For the introduced water‐equivalent material slab downstream mimicking AC beyond the target, we expected to measure the introduced WET shift with RP while we did not expect to measure it with PGI, as the delivered treatment field was not affected by the introduced material slab.

For the couch movements down or left in BEV, we expected similar, but not the same, range shifts for RP and PGI, as the spot positions of the plans were not the same and RP spots were also affected by tissue changes distal to the target. For the couch movement upstream, we expected PGI to measure a shift due to the system's fixed position in the treatment room relative to the patient. We did not expect to measure a shift with RP as the RP‐field was only marginally affected by a slightly smaller air gap.

The accuracy (*A*) of both the PGI and RP systems was determined as the difference between the weighted mean $\left(\hat{\mu }\right)$ of the measured range shifts ($M_{i}$), evaluated as the spot‐wise median of the ten times repeated field irradiation, and the ground truth (*G*):

(5) $$A=\hat{\mu }-G,$$

with

(6) $$\hat{\mu }=\frac{\sum_{i=1}^{N}w_{i}M_{i}}{\sum_{i=1}^{N}w_{i}},$$

where *N* is the total number of spots and the weights ($w_{i}$) are given by the inverse of the spot‐wise proton range variance ($1/\sigma_{i}^{2}$).

The corresponding precision was calculated as the weighted variance (${\hat{\sigma }}^{2}$) for each scenario:

(7) $${\hat{\sigma }}^{2}=\frac{\sum_{i=1}^{N}w_{i}}{{\left(\sum_{i=1}^{N}w_{i}\right)}^{2}-\sum_{i=1}^{N}{\left(w_{i}\right)}^{2}}\cdot \sum_{i=1}^{N}w_{i}{\left(M_{i}-\hat{\mu }\right)}^{2}$$

## 3 RESULTS

**FIGURE 3:**
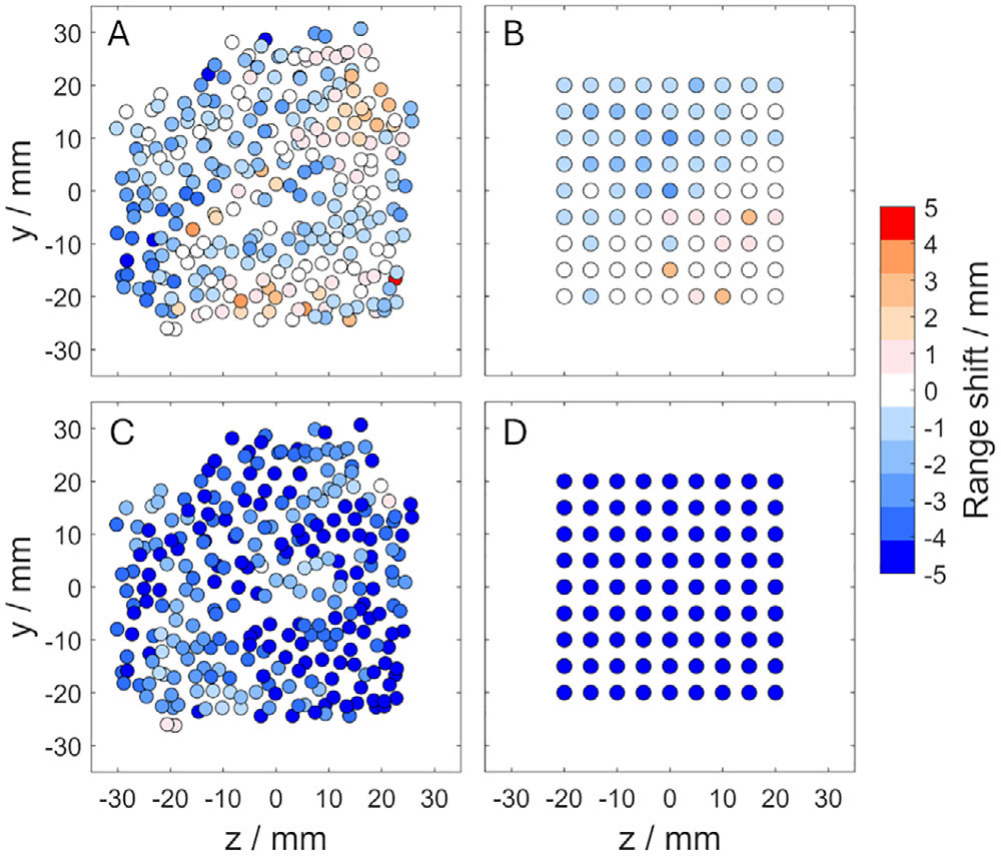
Local conformity between PGI and RP shifts shown as color maps in beam's eye view (BEV): (a) Detected PGI range shift (of the most distal spots) and (b) corresponding RP shift for a 3 mm downward couch movement in BEV. (c) Detected PGI range shift (of the most distal spots) and (d) corresponding RP shift for a 5 mm water‐equivalent material slab introduced into the clinical beam path. The center coordinate (*y* = 0, *z *= 0) represents the iso‐center of the respective plan. The *y*‐coordinate shows the vertical displacement in BEV (up/down), while the *z*‐coordinate represents the horizontal displacement in BEV (left/right). The coordinate system in respect to our setup is shown in Figure 2.

The local conformity between PGI and RP shifts in BEV for both a setup error and an induced anatomical change is illustrated in Figure 3. This visualization shows how the two verification methods correspond in identifying these variations, while also emphasizing their differences. The results for all scenarios investigated are presented in the following.

### 3.1 Introduced anatomical changes

**FIGURE 4:**
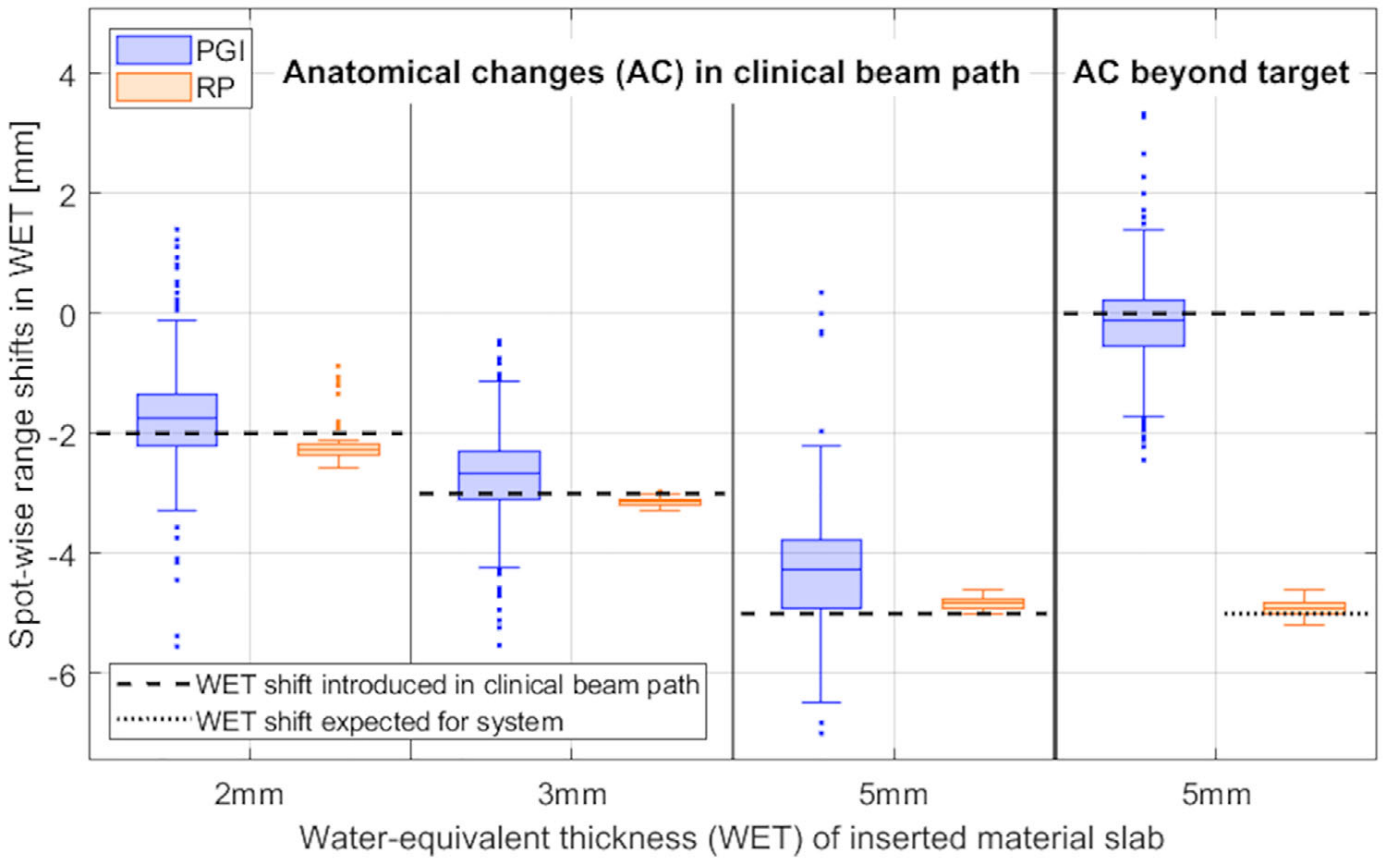
Detected spot‐wise range shifts in WET (median of 10 measurements) by prompt gamma imaging (PGI, blue) and range probing (RP, orange) for anatomical changes (AC) introduced by water‐equivalent material slabs (2, 3, and 5 mm) within the clinical beam path and a 5 mm slab beyond the target. Boxplots: The median over all spots is shown as a solid line, while the box edges represent the upper and lower quartile. The whiskers connect the minimum and maximum values that are no outliers, while outliers are more than 1.5 times the interquartile range away from the box and represented by single dots.

Both systems detected the introduced material slabs mimicking AC within the beam path as shown in Figure 4. RP measurements were more precise across all scenarios (RP: 1σ ≤ 0.3 mm, PGI: 1σ ≤ 0.8 mm). RP and PGI were similarly accurate for the 2 mm slab with their accuracy being within 0.2 mm. RP demonstrated higher accuracy for 3 and 5 mm slabs, while PGI slightly underestimated those slabs (accuracy of RP: within 0.2 mm, accuracy of PGI: within 0.7 mm). The material slab mimicking AC beyond the target, therefore not affecting the current treatment field, was only detected by the RP system with an accuracy being within 0.1 mm.

### 3.2 Introduced setup errors

**FIGURE 5:**
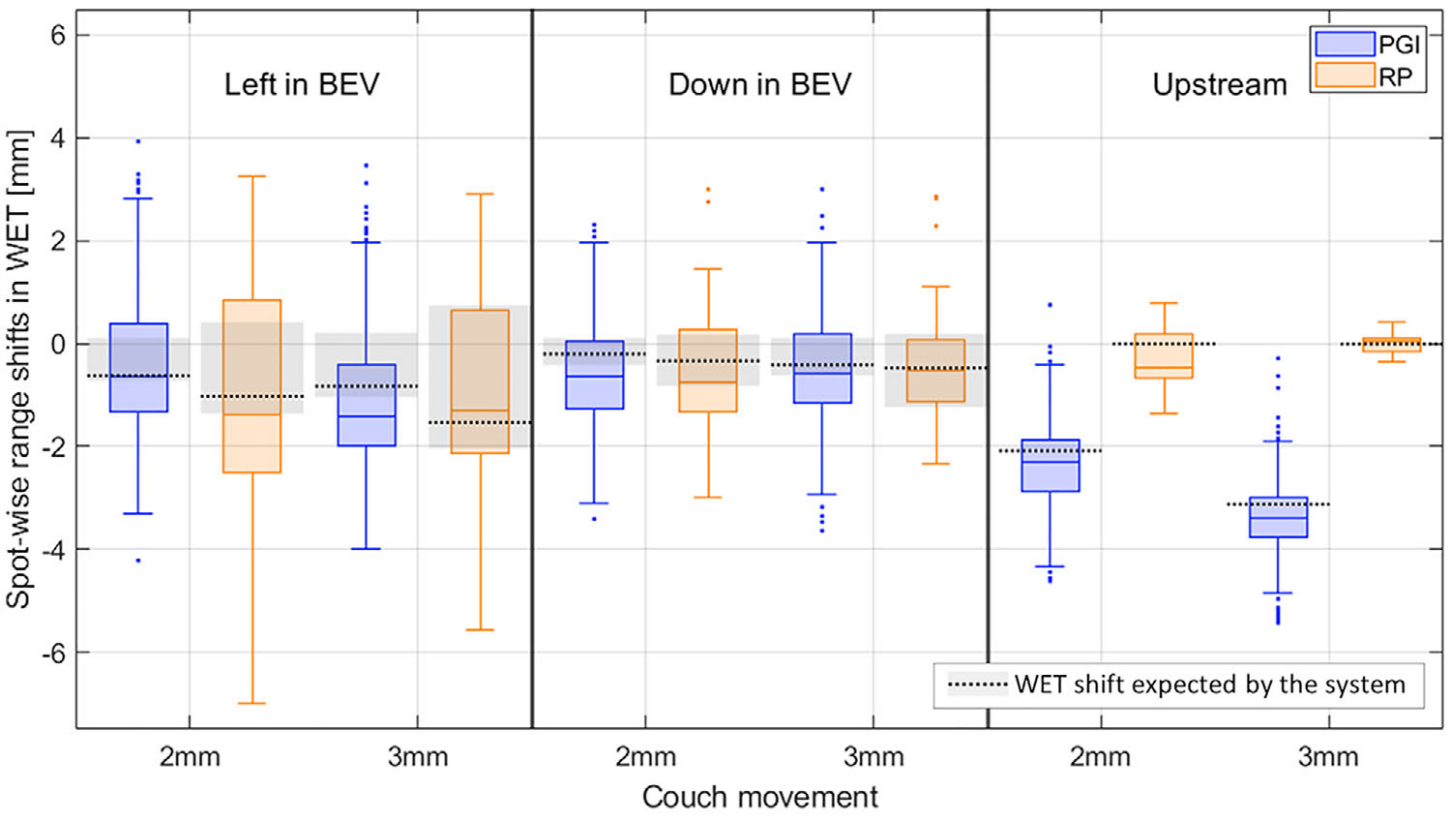
Detected spot‐wise range shifts in WET (median of 10 measurements) by prompt gamma imaging (PGI, blue) and range probing (RP, orange) for SE introduced through couch movements of 2 and 3 mm in three axial directions: left in BEV, down in BEV and upstream. The median of the expected range shifts for each system are represented by black dotted lines while the grey shaded areas include the upper and lower quartile. Boxplots are defined as in Figure 4.

Both systems detected their expected range shifts for all setup error scenarios, with RP being more accurate than PGI (accuracy of RP: within 0.3 mm, accuracy of PGI: within 0.8 mm). The couch movement left in BEV was detected with slightly better precision by the PGI system (RP: 1σ ≤ 1.0 mm, PGI: 1σ ≤ 0.9 mm), while the precision of detecting the couch movement down in BEV was the same for both systems (RP, PGI: 1σ ≤ 0.7 mm). The upstream couch movement was only recognized by PGI with an accuracy being within 0.4 mm (Figure 5).

## 4 DISCUSSION

For the first time, two different yet complementary treatment verification methods were directly compared experimentally: prompt gamma imaging and range probing. Both methods proved their feasibility to detect the different investigated treatment deviation scenarios, highlighting their potential for treatment verification in OAPT. Additionally, the compatibility of the two systems was successfully demonstrated in a clinically realistic setting.

While the quantitative accuracy of both methods was comparable, RP demonstrated greater precision than PGI. It is important to highlight that PGI samples the complete clinical treatment plan, while RP is currently technically limited to a sparser sampling. As a result, the RP‐field might, in some cases, fail to fully encompass the entire target volume, potentially missing treatment deviations occurring outside its coverage. However, in this study, complete coverage was achieved due to a small target and moreover, with increased IC diameter and further advanced techniques, such as flat panel detectors, it will be possible to monitor larger fields. Furthermore, RP detected an introduced anatomical change beyond the target, which did not influence the treatment field. RP does not provide information on the location in depth of detected anatomical changes, which limits its ability to distinguish between anatomical changes relevant and non‐relevant for the current treatment field, potentially leading to unnecessary interventions in some cases. However, since patients are typically treated with multiple fields, these anatomical changes could affect other treatment fields, and by detecting the changes in advance, appropriate measures could be taken. Moreover, relevant changes within the clinical beam path may go undetected if they are compensated beyond the target. Additionally, the application of RP is limited for beams oriented from superior to inferior and becomes infeasible when the highest‐energy protons do not exit the patient, as they cannot be captured by the MLIC.

The effect of introduced couch movements on range deviations heavily depends on the patients’ anatomy. Defining a ground truth for the actual range deviation is therefore difficult and must be done for each system individually. Furthermore, couch movements are inherently affected by submillimeter uncertainties of the positioning system which could have potentially influenced the results. Despite these limitations, both systems were able to detect the introduced couch movements, mimicking setup errors relevant to the treatment field. PGI also detected couch movements in beam direction, which have a vanishing influence on the relevant treatment field. However, in case of multiple fields, the setup error might become relevant and could be adjusted before delivering subsequent fields.

The minimum MU that could be delivered at OncoRay resulted in an administered dose of approximately 3 cGy per RP‐plan, while at UMCG the minimum MUs result in a dose of only 1cGy per RP‐plan.^19^ Although this was not problematic for a head phantom, if the technique was to be implemented in patients in the future, based on the ALARA principle, the lowest possible dose must be delivered and potentially the RP dose could be even incorporated into the treatment plan directly.

Our results for detecting anatomical changes agree with another study where PGI was compared to prompt gamma spectroscopy (PGS) using the same anthropomorphic head phantom. This study investigated and compared the accuracy and precision of PGI and PGS when introducing material slabs covering half of the treatment field to mimic anatomical changes. While that comparison was conducted at two different proton facilities, requiring careful cross‐calibration and alignment of the measurement setup, treatment plans, and experiment protocols,^27^ our study was performed as a simultaneous experiment, benefiting from the same treatment planning and delivery system.

PGI enables in vivo treatment verification during field delivery without adding dose to the patient nor prolonging the treatment. PGI has reliably validated the CT‐based proton range prediction at OncoRay.^23^ Furthermore, PGI has shown to reliably classify relevant treatment deviations in retrospective evaluation of its clinical application by processing complex input data through both artificial intelligence and analytical classification methods.^9^, ^30^ Moreover, it was shown that the use of PGI for online treatment verification allows the reduction of current range uncertainty margins.^11^ The next step is to implement PGI‐triggered treatment interventions in an upcoming clinical trial at OncoRay, where patients will benefit from reduced safety margins.^11^

RP enables in vivo verification of the proton beam range prior to treatment delivery. RP has reliably validated the CT‐based range prediction at UMCG^31^ and verified the accuracy of CT numbers in CBCT‐based synthetic CT images.^32^ RP‐QC technique has demonstrated its capability to assess range accuracy for head‐and‐neck patients, potentially enabling a reduction in safety margins.^33^ In this study, measurement loading, range error computation, and error‐map visualization were completed in an average of 5 seconds, a processing time sufficient for the use of RP in OAPT. Future steps will focus on extendin*g in vivo* RP‐QC to additional tumor indications and integrating RP‐based verification into OAPT workflows, thereby facilitating adaptive clinical decision‐making.

First OAPT workflows are currently finding their way into clinical practice.^34^ So far, they are based on daily in‐room CTs, which are used for plan adaptations to correct for inter‐fractional anatomical changes and setup errors.^2^ Using PGI for online treatment verification would enhance current workflows by triggering adaptations also for intra‐fractional changes, thus paving the way for (near) real‐time adaptive proton therapy.^35^, ^36^

OAPT workflows could also be used with CBCT scanners, which are widely available in proton therapy facilities. However, CBCTs increase the uncertainty in determined CT numbers, requiring an additional safety margin and, even more important, especially in the early phase of introduction, an additional safety net. RP has demonstrated its effectiveness in evaluating the accuracy of CT numbers in CBCT‐based synthetic CT images,^21^, ^22^ thereby serving as a critical safety net for addressing potential treatment deviations and artifacts in sCT generation before the start of the treatment. Furthermore, a recent study has shown that also the PGI system can serve as complimentary safety net in CBCT‐based OAPT during delivery.^12^ Hence, with CBCTs and corresponding treatment verification systems, a more widespread application of OAPT would become possible.

Using the same experimental setup for both verification methods, our study demonstrated for a horizontal beam angle that these approaches are compatible and can seamlessly coexist within a clinical environment—a setup that could also be incorporated into a gantry system. This integration allows valuable information to be obtained both prior to treatment (from RP) and during treatment delivery (from PGI). RP offers highly precise and accurate detection of deviations in proton range or artifacts in sCT generation within the RP field prior to treatment. This gives the possibility to correct them before the patient is treated. However, RP cannot distinguish whether detected anatomical changes impact the treatment field. It fails to detect unexpected deviations during treatment as well as changes outside the RP field and it requires additional radiation exposure for its measurement. Conversely, PGI offers precise and accurate detection of anatomical changes during field delivery, distinguishing relevant from non‐relevant changes for the monitored treatment field without adding dose or extending treatment time. However, changes detected by PGI cannot be accounted for prior to treatment. Each system independently provides essential and robust safety‐net functionalities for OAPT. However, in certain situations, for example, when a monitoring before and during the treatment is needed, a combined use would be valuable to leverage each system's full range of advantages.

## 5 CONCLUSIONS

PGI and RP precisely detected introduced anatomical changes and setup errors relevant for the current treatment field. RP allows for pre‐treatment range verification after setup imaging using a low‐dose RP‐field, while PGI provides treatment verification during field delivery by detecting range deviations relevant to the treatment field. By setting up PGI and RP simultaneously, the study demonstrated their compatibility, as well as the unique advantages of each system and their crucial role as safety nets in OAPT.

## CONFLICT OF INTEREST STATEMENT

OncoRay and UMCG have institutional research agreements with Ion Beam Applications S.A. (IBA). G. Janssens is an employee of IBA. OncoRay also has an institutional research agreement with Siemens Healthineers in the field of CT imaging for particle therapy. For the present study, the authors received no financial support, neither for the design of the study or the selection of the materials used, nor for the collection, analysis and interpretation of the data nor for the preparation of the publication. The authors report no conflict of interest.

## Data Availability

Authors will share data upon request to the corresponding author.
